# Draft genome sequence of *Arthrobacter* sp. strain B10-11 isolated from the tomato rhizosphere

**DOI:** 10.1128/mra.01220-23

**Published:** 2024-03-22

**Authors:** Ruben Chaboy-Cansado, Paula Cobeta, Gabriel Roscales, Daniel Aguirre de Cárcer, Alberto Rastrojo

**Affiliations:** 1 Departamento de Biología, Universidad Autónoma de Madrid, Madrid, Spain; University of Strathclyde, Glasgow, United Kingdom

**Keywords:** *Arthrobacter*, rhizosphere, soil, genome

## Abstract

In the present work, we present the draft genome sequence of a new putative *Arthrobacter* species associated with the tomato rhizosphere.

## ANNOUNCEMENT

*Arthrobacter*, belonging to the *Micrococcaceae* family within the *Actinobacteria* phylum, is a genus of obligate aerobic Gram-positive bacteria commonly found in soil and in association with plant rhizospheres (1–3). The genus was proposed by Conn and Dimmick showing a rod morphology while growing exponentially and cocci morphology in stationary cultures (4, 5). Members of the *Arthrobacter* genus are gaining interest due to its potential use in burned soil bioremedation or its ability to enhance nutrient availability and alleviate salt stress in plants (6–8). Here, we present the draft genome sequence of *Arthrobacter* sp. strain B10-11 isolated from the tomato rhizosphere.

Tomato seeds (Salinas F1, CapGen) were sterilized by immersion in 70% ethanol and later 10% bleach and then germinated in agar plates for 2 days at 28°C in the darkness. Germinated seeds were planted under sterile conditions in a substrate composed of 3 g of perlite and 3 g of soil from a backyard garden from Madrid (Lat:40.496480N, Lon:3.689131 W). After 3 weeks of growth in a controlled greenhouse (24°C for 16 hours with light and 18°C for 8 hours in the darkness), roots were harvested removing soil and perlite and then immersing in PBS buffer. After vortexing, roots were removed using sterile tweezers and the resulting microbial suspension was serially diluted and inoculated onto R2A agar plates supplemented with cycloheximide (100 µg/mL) to avoid fungal growth. After 24–48 hours at 28°C, bacterial colonies were picked and re-isolated by streaking onto new plates three times. The selected bacterial colony was grown in liquid media (R2A), and genomic DNA was extracted using the NucleoSpin Microbial DNA kit (MACHEREY-NAGEL). Sequencing library was prepared using the Nextera XT Library Prep kit (Illumina) and sequenced in a MiSeq device (Illumina) using a MiSeq Reagent Kit v2 (500 cycles). Unless otherwise noted, default parameters were used for all software. A total of 1,372,262 pairs of reads (2 × 250 bp) were obtained. After read quality trimming with Trimmomatic (v0.30) (9) 1,309,686 pairs of reads remained. A total of 4,534,959 bp were assembled (11 contigs longer than 5 kb, N50 = 700,218 bp and GC% = 65.98) using SPAdes (v3.15) (10), which, according to MiGA (v1.2.18.2) (11), is 97.2% complete, having a complete tRNA gene set (50 genes) for all 20 canonical amino acids and all ribosomal RNA genes (one 16S copy, three 5S copies, and one 23S partial copy). Genome annotation was carried out using NCBI’s Prokaryotic Genome Annotation Pipeline (PGAP, v6.6), which found 4,162 total genes (97.8% protein-coding genes). Whole genome analysis against all available *Arthrobacter* genomes in GenBank (by Oct-23) using FastANI (v1.1) (12) showed that the closest type strain is *Arthrobacter oryzae* strain AO2021 (with accession GCF_030718995.1 and 96.3% ANI). However, *A. oryzae* strain AO2021 only shared 82.2% ANI with *A. oryzae* reference strain DSM 25586 (genome accession GCA_003634095), which is under the commonly considered bacterial species demarcation criterion (95%–96%) (13). Therefore, we believed that the strain may belong to a new *Arthrobacter* species, together with strain AO2021, an idea also supported by MiGA’s determination of new species test (*P* value: 0.019).

## Data Availability

The genome sequence has been deposited at NCBI under BioProject PRJNA1024081 and BioSample SAMN37765996. Trimmed Illumina reads can be found under SRA run accession number SRR26498647 and the assembled genome at GenBank with accession number JAWMTH000000000.
